# EPNs Exhibit Repulsion to Prenol in Pluronic Gel Assays

**DOI:** 10.3390/insects11080457

**Published:** 2020-07-22

**Authors:** Tiffany Baiocchi, Chunjie Li, Adler R. Dillman

**Affiliations:** 1 Department of Nematology, University of California, Riverside, CA 92521, USA; tbaio001@ucr.edu; 2 Key Laboratory of Mollisols Agroecology, Northeast Institute of Geography and Agroecology, Chinese Academy of Sciences, Harbin 150081, China; lichunjie@iga.ac.cn

**Keywords:** entomopathogenic nematodes, PF127, pluronic gel, chemotaxis

## Abstract

Entomopathogenic nematodes (EPNs) are lethal parasites of insects that have become valuable in biological control and as a model system for studying host–parasite interactions, behavioral ecology, neurobiology, and genomics, among other fields. Their ability to locate hosts is paramount to successful infection and host seeking has been extensively studied in many species in the lab. Here, we explored the usefulness of pluronic gel as a medium to assess EPN host seeking in the lab by characterizing the response of *Steinernema carpocapsae*, *S. feltiae*, *S. glaseri*, *S. riobrave*, *Heterorhabditis bacteriophora*, and *H. indica* to the odor prenol. We found that the infective juveniles (IJs) of these species were repelled by prenol in pluronic gel. We then evaluated how storing the IJs of *S. carpocapsae*, *S. feltiae*, and *S. glaseri* for different amounts of time affected their behavioral responses to prenol. The response of *S. carpocapsae* was significantly affected by the storage time, while the responses of *S. feltiae* and *S. glaseri* were unaffected. Our data support the notion that pluronic gel is a useful medium for studying EPN behavior and that the response of *S. carpocapsae* to informative odors is significantly affected by long-term storage.

## 1. Introduction

The insect-killing parasitic nematodes, known as entomopathogenic nematodes (EPNs), have been leveraged as biological control agents for use in home gardens and industrial agriculture, and are used as models for studying host–parasite interactions [1,2]. In the EPN life cycle, the infective juvenile (IJ), a developmentally arrested stage, and the only free-living stage, leaves a depleted host to seek out a new one in order to resume its development. A variety of cues such as temperature [3], tactile [4] and olfactory [5,6,7], play a role in, and impact, the host-seeking process. Chemoreception in particular is leveraged by the nematodes to locate and infect hosts [4], and as such has been the focus of many EPN behavioral studies. Such studies have often aimed to characterize which odors EPNs use as informational cues [5,6,7], as well as how these odors are used by the EPNs to perform the host-seeking (or avoidance) behaviors that are essential to their survival.

Efforts in studying EPN behavior over the last 60 years have primarily relied on three main forms of in-lab behavioral assays: those done in sand or soil (which provide the best imitation of the natural environment), those done on agar-based media, and—more recently—assays that employ the use of pluronic gel (PF127) media [8]. PF127 is a hydrogel made of hydrophilic polymers capable of holding large amounts of water and forming three-dimensional polymeric networks. There are many biomedical applications for this substance including its use as a drug carrier. One of its most useful properties is that it is liquid at low temperatures, but transitions to a gel state at higher temperatures, such as body temperature. The temperature at which the transition from liquid to gel occurs is dependent on the concentration of the media [9].

Although in-field experiments may be the best experimental format for assessing the efficacy of EPNs, in vitro behavioral assays play an important role in informing EPN behavioral decision making. Details uncovered through laboratory-based behavioral assays include: which odors play a role in host seeking (or avoidance), to what extent certain odors influence host seeking (or avoidance), and the hierarchy of decision making based on olfaction [3,5,6,7,10,11]. In vitro behavioral assays have been successfully used to reveal important features of host-seeking behaviors in EPNs.

Sand-based and agar-based media have both been used to discover some important aspects of EPN biology, but each comes with advantages and disadvantages [8]. For instance, sand or soil-based assays replicate the most natural environment that the EPNs may experience and may provide the best method that replicates natural EPN behaviors. Soil-based assays provide a matrix in which EPNs can move freely in three-dimensions and potentially provide tactile feedback as IJs move through the media and may even introduce the complexity of soil-based odors that would naturally occur in the field. However, these benefits come at the cost of difficulty in the observation, measurement, and (potentially) the set-up (or resetting) of the assay arena. Of particular concern is the difficulty with the observability and measurement, since the measurement requires the collection of soil and the separation of IJs from soil particles in order to be observed. This can make it difficult and impractical to accurately evaluate the behaviors. Furthermore, the accuracy of measurements relies heavily on the ability of researchers to recover all the IJs from the soil, a time-consuming process that still relies on the assumption that all IJs are recovered when the measurements are taken. 

Although agar-based assays lack the allowance for three-dimensional movement as well as the other complexities offered by soil-based assays, there are several benefits to running behavioral assays using agar media. Agar-based behavioral assays are easier and quicker to set up (compared to some soil-based assay designs) and control. Additionally, agar-based chemosensory assays provide an arena in which the IJs are easily observable and can be accurately quantified without the assumption of whether or not all the IJs are visible. Furthermore, the measurements can be repeatedly taken over time without greatly disrupting the assay arena [12]. 

PF127 media provides an easily traversable matrix in which nematodes can move in three dimensions and potentially experience similar tactile sensations that would be experienced while traversing through a medium-like soil. Moreover, since the media is clear, behavioral assays in this medium are observable, similar to agar-based assays. The data acquired in PF127 allow for accurate methods for taking measurements without disrupting the assay arena. Because of these qualities, PF127 has been extensively used in plant-parasitic nematode (PPN) research, to evaluate the behavioral responses of PPNs to stimuli such as root exudates [13,14]. Despite the use of PF127 in the research on PPNs, its use in EPN behavioral research has been recent and limited; having only been used to evaluate root exudates, pH and volatiles released by gnat larvae [15,16]. 

Although PF127 is promising as an assay medium for EPNs, there are a few notable differences that need to be considered and evaluated. For instance, the starting temperature is different between assays on agar (which start and run at room temperature) and PF127 (in which nematodes are suspended while the solution is semi solid at room temperature and liquid at or below 15 °C). In addition, the effects of moving through the PF127 matrix (rather than along the surface of the media as occurs in agar-based assays) may also impact the overall responses to host-associated cues. Exploring these effects requires using an odor or stimulus that induces a robust response that has been previously studied. We selected prenol (3-methy-2-butene-1-ol) as an ecologically-relevant odor that is associated with EPN-infected *Galleria mellonella* and has been shown, over multiple studies, to elicit aversion behavior in EPN IJs [5,17,18]. Using PF127 and a modified assay design, we evaluated EPN IJ responses to the odor prenol. We then investigated the ability of EPNs to withstand long storage periods and the effects this had on chemotaxis behavior. Effective host seeking (or avoidance) is essential to EPN behavior, even after they have been stored for long durations. We hypothesized that the behavioral responses would be similar in PF127 as they were in agar- and sand-based assays, and that with increased storage time, the IJs would be more likely to ignore prenol as a deterrent cue. In this study, we sought to provide a comparison that could demonstrate any impacts using PF127 as an assay medium might have on the behavioral responses of EPN IJs in response to a specific odor, as well as the impacts of storage time on chemotaxis behavior.

## 2. Materials and Methods

### 2.1. Animal Care

Entomopathogenic nematodes *Steinernema riobrave* (strain TX-355), *Steinernema glaseri* (strain NC), *Steinernema carpocapsae* (All), *Steinernema feltiae* (strain SN), *Heterorhabditis bacteriophora* (strain m31e), and *Heterorhabditis indica* (strain Hom1), were cultured using standard protocols as previously described [5,19]. After being collected from white traps and rinsed, the IJs were then diluted to approximately 1000 IJs/mL with tap water, and held in 75 cm^2^ cell culture flasks, while being stored at 15 °C to help prevent the cold shock upon the introduction to the PF127 media. The IJs were stored at this temperature for 7–14 days before use in the dose–response assays, and up to 155 days for the assays evaluating the effect of storage on behavior. 

### 2.2. Pluronic Gel Behavioral Assay

The PF127 was made as previously described [16,20]. A clean stir bar was added to a flask or bottle of an appropriate size. Forty milliliters (half the total volume of water to be used) of sterilized MilliQ water was added to a glass flask or bottle, 23 g of pluronic f-127 powder was added to the flask followed by a remaining 40 mL of water (second half of total volume of water). The container was placed on a stir plate and left at 4 °C overnight to mix. This resulted in approximately 23% PF127 solution.

**Figure 1:**
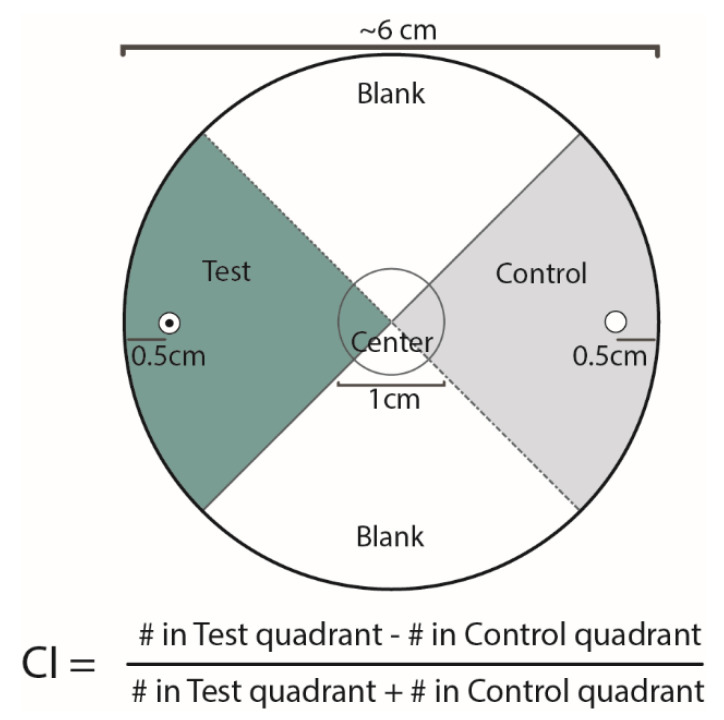
Scoring template for the PF127 gel assay. Assays were done using 60 mm petri dishes filled with approximately 10 mL of prepared PF127. Five microliters of the test compound (prenol) was placed within the test region at the designated position, while 5 μL of the control (diluent) was placed within the control region at the designated position. Approximately 100 infective juveniles (IJs) (which had been stored at 15 °C prior to the assays) were placed just under the surface of the media (at the center) before it solidified at room temperature, leaving the IJs embedded in the PF127 3-dimensional matrix during the assay, unless they moved to the surface on their own. Assays were run at room temperature, approximately 23 °C before being scored. Chemotaxis index (CI) is calculated as shown.

To prepare the assay arenas, sterile 6 cm petri dishes were prepared by adding a quadrant assay template to the bottom of each dish [20] (Figure 1). Approximately 100 IJs were pelleted by allowing them to settle in a 1.5 mL tube while the plates were prepared. Then, 10 mL of the prepared PF127 solution was carefully added to each 6 cm plate, to avoid introducing any bubbles to the solution. The 100 EPN IJs were extracted from the tube in a volume of 5 µL and placed into the center of each assay’s plates just under the surface of the gel.

Two opposing quadrants were left blank during the assay. For the other two opposing quadrants: one received 5 µL of 100% ethanol (our diluent and control), and the diametrically opposed quadrant then received 5 µL of prenol (diluted in 100% ethanol to the respective concentrations ranging from 2 M to 2 mM). Both the control and odorant were placed just below the surface of the PF127 media. The IJs of each EPN species within each region were counted each hour to determine how long to run the assay for each species. When the number of IJs in the test and control region stabilized at the subsequent time point (i.e., did not change from one hour to the next), the time point of stabilization was chosen as the assay time for that species. 

Because the IJs of each species disperse and move at different rates, the assay time for each species was adjusted to account for these differences. Assays for *S. glaseri*, *S. riobrave* and *H. indica* were run for 2 h before being scored. *S. feltiae* and *H. bacteriophora* were scored at 1 h. *S. carpocapsae* was allowed to run for 6 h due to its characterization as an ambusher that displays low participation and slow dispersal capabilities [21,22].

Scoring plates was done using a transillumination stereoscope. Within the test and control quadrants, the entire depth of the media was evaluated and each nematode within these regions (outside of the circle around the initial placement zone) was counted and used for determining the chemotaxis index (Figure 1). In addition to calculating the chemotaxis index (CI), we also determined and compared the participation of the nematodes in chemotaxis assays. This was measured as the proportion of the population of IJs that remained in the center of the plate depicted in Figure 1, and that did not participate in the assay by migrating to any of the other defined quadrants of the assay arena.

### 2.3. Experimental Replicates and Normalization

**Figure 2:**
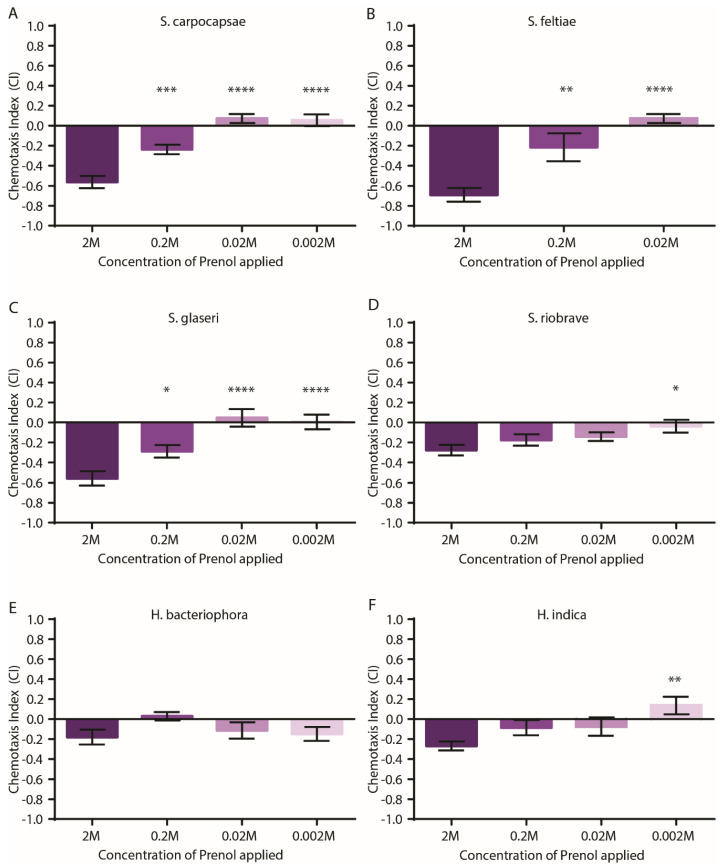
Responses of the entomopathogenic nematode (EPN) infective juveniles (IJs) to varying doses of prenol in the PF127 media chemotaxis assay. For the chemotaxis index (CI) values, a score near −1 indicates a high aversion, near 0 indicates neutrality, and +1 indicates a strong attraction. The concentrations listed along the *x* axis (of all graphs) represent the initial concentration of the compound applied to the plate (5 µL applied to the test region and 5 µL of diluent (ethanol) applied to the control region). Chemotaxis responses by (**A**) *S. carpocapsae*, (**B**) *S. feltiae*, (**C**) *S. glaseri*, (**D**) *S. riobrave*, (**E**) *H. bacteriophora*, and (**F**) *H. indica.* Bars represent the mean with error bars representing SEM. For all the graphs, a one-way ANOVA with Dunnett’s multiple comparisons post-test (comparing the value for the response to 2 M to all other concentrations tested) was used. * *p* < 0.05, ** *p* < 0.01, *** *p* < 0.001; **** *p* < 0.0001. Experiments consisted of 4 plates for an experiment, with 3 biological replicates.

The dose–response curve experiments (Figure 2) consisted of four (in-parallel) technical replicates per experiment (biological replicate). For *S. carpocapsae*, *S. feltiae*, and *S. glaseri*, a total of two experiments (biological replicates) were performed (for a total of 8 replicates for each dose tested). For *H. indica, H. bacteriophora*, and *S. riobrave*, a total of three experiments (biological replicates) were performed (for a total of 12 replicates for each dose tested for each species).

**Figure 3:**
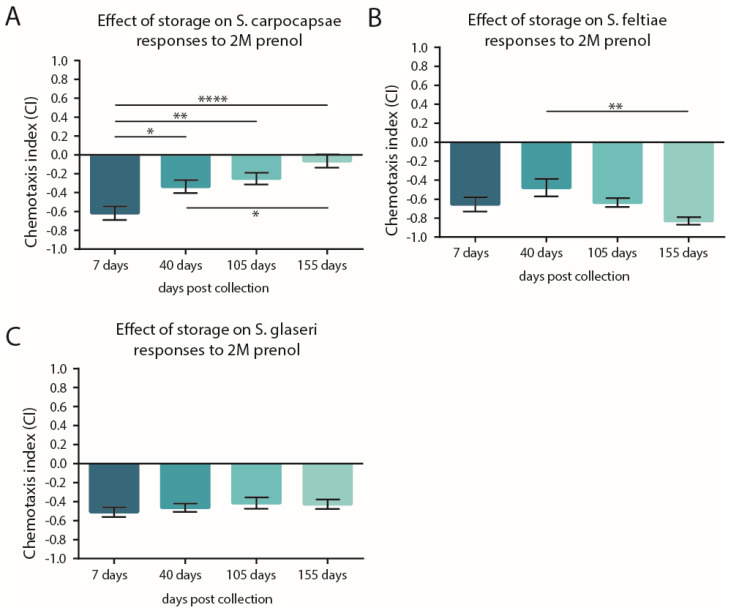
Effects of the storage-time on the responses of the *Steinernema* infective juveniles (IJs) to 2 M prenol. For the chemotaxis index (CI) values, a score near −1 indicates a high aversion, near 0 indicates neutrality, and +1 indicates a strong attraction. Three species of *Steinernema* were selected and the IJs were stored at 15 °C for the specified durations (7 days, 40 days, 105 days, and 155 days) prior to assessing their response to prenol using the PF127 assay medium. The assays were run for 2 h for *S. glaseri* before being scored, 1 h for *S. feltiae*, and 6 h for *S. carpocapsae*. Movement into the test and control regions of the assay were used to calculate the CI. (**A**) The responses of (**A**) *S. carpocapsae* IJs, (**B**) *S. feltiae* IJs, and (**C**) *S. glaseri* IJs. Bars represented the mean with error bars, representing the SEM. Statistical analysis was done using a one-way ANOVA with Tukey’s multiple comparisons post-test; comparing the values to the responses at 7 days post collection. * *p* < 0.05, ** *p* < 0.01, **** *p* < 0.0001.

For evaluating the effect of storage time (Figure 3), each experiment (biological replicate) consisted of four (in-parallel) technical replicates. *S. glaseri* yielded varying results for the first time point tested and thus four experiments were performed (for the initial and all subsequent timepoints) to account for the variability in the *S. glaseri* behavioral data. This resulted in a total of 16 replicates for each timepoint tested for this species. For *S. carpocapsae* and *S. feltiae*, two experiments (biological replicates) were performed for a total of 8 replicates per storage time evaluated. 

The replicate numbers varied between species, but within a species the number of replicates across the conditions (either storage time or dose–response) was consistent. However, to make the cross-species evaluation possible, we normalized the data in the following ways.

For the cross-species evaluation regarding the dose–response curve experiments (Supplementary Figure S1), we normalized the data to a total of 6 total replicates. For the species with two experiments (biological replicates), we selected the first three (in-parallel, technical) replicates from each experimental set. For the species with three experiments, we selected the first two (in-parallel, technical) replicates. This seemed like an unbiased way of normalizing the data to compare across the species for the responses to varying doses of prenol.

For the cross-species evaluations of storage-time effect experiments (Supplementary Figure S2), we only normalized the values for *S. glaseri* (since we performed four experiments instead of only two). To normalize the *S. glaseri* to a total of 8 replicates (so it would match the number of replicates for the other two species) we selected the first two (in-parallel, technical) replicates from each experiment at each timepoint. 

### 2.4. Statistical Analysis

All the statistical analysis was done using GraphPad PRISM software. Analyses of the behavioral responses (for each species) to varying doses of prenol used a one-way ANOVA with Dunnett’s multiple comparisons post-test. These analyses were performed comparing the response of the nematodes to 2 M with all the other concentrations tested. This was done to identify any statistically significant shift in the behavioral response between the doses (Figure 2). 

To assess the effect of EPN storage time on the response to 2 M prenol, a one-way ANOVA with Tukey’s multiple comparisons post-test was used. These analyses compared the results of each post-collection time point (i.e., the days the IJs were stored, which could also be considered age) to all other post-collection time points (Figure 3). 

For the analysis of the participation values pertaining to the behavioral responses (of each species) to varying doses of prenol, a two-way ANOVA was used with Dunnett’s multiple comparison post-test, comparing the responses to all doses with those in response to the 2 M dose (Supplementary Figure S3). 

The analysis of the participation data from the storage-effect assays utilized a two-way ANOVA with a Dunnett’s multiple comparisons post-test. Participation was calculated as the proportion of nematodes that migrated to one of the four quadrants (towards test, towards control, towards blank, or center). The statistical analysis on participation compared all the values within each category to the response to 2 M. 

For the participation data in Supplementary Figure S4, a two-way ANOVA with Dunnett’s multiple comparisons post-test was used to compare the responses of each population’s age (40, 105 and 155 days post-collection) with the responses at the earliest timepoint (7 days). This comparison was done within each category of “test quadrant”, “control quadrant”, “blank quadrants x2”, or “center”.

For the cross-species comparisons, the statistical analyses were as follows: the dose–response assay data were normalized as described above in Section 2.3. For the proportions that remained in the center (Supplementary Figure S1A) and the proportion that was in the blank quadrants (Supplementary Figure S1B): a two-way ANOVA with Tukey’s comparison post-test was used to compare all the species to one another within each dose category, with the exception of the 0.002 M dose—for which the *S. feltiae* data had not been collected, yielding an error in the GraphPad PRISM software that would not allow a two-way ANOVA to be done without this included). To compensate, we performed an additional two-way ANOVA with Tukey’s multiple comparisons post-test, after removing the *S. feltiae* data and comparing the remaining species responses to one another for all the doses including the 0.002 M dose (statistical results for the higher doses remained unchanged when the *S. feltiae* data was removed). 

For the cross-species evaluation of storage time effects on responses, the data for the *S. glaseri* was normalized as described in Section 2.3. Statistical analysis was done using a two-way ANOVA with Tukey’s multiple comparisons post-test, comparing between the responses of each species within the designated time points (of 7 days, 40 days, 105 days or 155 days) (Supplementary Figure S2).

## 3. Results

### 3.1. EPNs Are Repelled by Prenol in Pluronic Gel Assays

Previous research using standard chemotaxis media has shown that EPNs are repelled by prenol as a distal informational cue [5,18]. Wanting to establish the PF127 media as a viable option for introducing a three-dimensional matrix to test EPN behavior, we leveraged prenol as a strong chemotactic cue in the PF127 media assay. The results revealed that EPNs still initiated repulsion behavior when exposed to prenol, and that the response to the odor waned with decreasing concentrations of prenol applied to the assay (Figure 2). However, for some species (*S. riobrave* and *H. indica*) the repulsion responses to prenol—even the highest doses—were mild (Figure 2). Overall, these data demonstrate that even within a three-dimensional matrix (as opposed to the nematodes traversing across the surface of an agarose media) the EPNs still detect and respond to prenol as a distal cue across multiple EPN species and even across two genera. Among the species tested, *H. bacteriophora* was the only species that did not have a significant shift in behavior in response to the decreasing dose of prenol applied to the plate, although it did show a mild repulsive behavior to multiple doses (Figure 2E). 

Despite the variation in the CI values between species, the participation observed appears not to vary greatly from dose to dose or even between species (Supplementary Figures S1 and S3). It is worth noting that the participation of the IJs moving towards the test quadrant reflects the results reported by the CI values (Figure 2). Furthermore, the participation data revealed that for all the species and doses (with the exception of *S. riobrave* at the 0.002 M dose), less than 50% of the population stayed within the initial placement—i.e., 50% of the population was moving out of the center and participating in the assay. In addition, even at the lowest dose used for each species, approximately 32–40% were contributing directly to the CI value calculation.

### 3.2. Storage Time May Not Shift the CI but Can Impact Participation

In agricultural use, it is not uncommon for EPNs to endure extended storage times. It has been previously demonstrated that storage temperature may affect both the survival and infectivity of EPNs [23,24]. Since previous work had demonstrated that the response of some EPNs to prenol were affected by moderate storage times [5,18], we wondered how longer time periods of storage/aging would impact the IJs’ behavior. To evaluate the effects of long-term storage on the response to prenol, we leveraged the PF127 assay to test the EPNs held at 15 °C over the course of a few weeks to several months. The results revealed that of the three *Steinernema* species tested, only *S. carpocapsae* experienced a significant loss of repulsion behavior (Figure 3). *S. glaseri* experienced no shift in its repulsion behavior, while *S. feltiae* actually exhibited its strongest repulsion behavior at the latest storage time point tested. 

The corresponding participation data of these storage assays (Supplementary Figures S2 and S4) also revealed that long-term storage did significantly affect participation. For both *S. feltiae* and *S. glaseri*, there was a significant loss of participation (increase in the proportion remaining in the middle), between the 7 day and 40 day time points, while *S. carpocapsae* exhibited a significant decrease in participation only after 155 days in storage.

## 4. Discussion 

The study of EPN chemotaxis behavior has predominantly utilized an assay setup, in which infective juveniles (IJs) move laterally along the surface of an agar-based chemotaxis media [3,7,18,25,26,27]. Although this has proven to be an effective assay design, it lacks the dynamic of three-dimensional motility that IJs would experience in a more natural environment like soil. Although some studies have used soil or sand to provide a more dynamic environment for the IJs to chemotax through [22,28], it increases the difficulty of taking accurate measurements since nematodes must be rinsed from the soil particles in order to be counted, while simultaneously preventing the direct observation of the behavior. The use of PF127 media provides both a semi-solid, three-dimensional matrix in which nematodes can easily move around while having the benefit of being clear and easily prepared in a lab for use in behavioral assays. For these reasons (among others), PF127 has been leveraged extensively in the research of PPN biology [13,29,30]. However, recent efforts have begun to further investigate the usefulness of PF127 media for the potential study of other nematode species such as EPNs. There are a few key differences between the conventional chemotaxis assay (using agar) and PF127 assays that are worth noting, and may account for the differences between our results and previous work that has evaluated the EPN response to prenol. 

For instance, the three-dimensional movement—a key feature provided in PF127 media—may impart potential drag against nematodes, which may hinder or slow their movements. To try and account for slower movement, the length of the assay can be increased as it has been in studies for PPNs [29] and even in EPN assays that leverage sand as a three-dimensional media [28]. However, in standard agar-based chemotaxis assays, the movement is less restricted in terms of drag, since nematode movements are across the surface of the media. It should be noted that, similar to agar-based assays, the concentration applied to the PF127 media is likely much higher than the concentration experienced by the nematodes, as the compound diffuses into media and may volatize into the air.

The effects of temperature should also be noted. Since the PF127 media goes from liquid to semi-solid as temperature increases (solidifying above approximately 15 °C), the nematodes are often added before the gel is solid (at temperatures lower than 20 °C), so that they are suspended within the matrix to allow for three-dimensional movement and host-seeking behavior. Temperature can affect EPN IJ behavior, even causing behaviors to switch entirely in some cases (i.e., attractive to repulsive or vice versa), as has been seen for the *S. carpocapsae* responses to p-cresol, 2-butanone and several other host-related odorants [3]. This may explain why the magnitude of the repulsion response was lower than in previous studies that used agar-based chemotaxis assays. However, the overall response across the EPNs tested revealed that most of the EPNs were still repelled by prenol, congruent with agar-based assays. 

There are some particular parallels that can be drawn between the responses seen in the PF127 assays utilized in this study and those seen in the studies using agar-based media. The first is that the CI values in response to prenol are similar to those shown in previous studies, which have revealed that prenol is a repulsive chemotactic cue for several EPN species [5,17,18]. However, it is worth noting that in previous studies, which used agar-based assays, EPN IJs responded more strongly to prenol, yielding lower CI values (more negative) than we observed using PF127 media. This may be due to a number of factors, including the difficulty of moving through the media (as opposed to moving along the surface) or even the nematodes’ capabilities of detecting prenol as a soluble cue as opposed to a volatized (airborne) cue. The difference in CI and the possible explanations we have reported here should be considered in future studies. 

In addition to finding that the general response to prenol was congruent with the data from agar-based assays, the corresponding participation data reflect the findings by the CI values; similar to previous EPN behavioral work that has measured participation (i.e., participation reflects movement towards the control region of the plate) [5,17,18]. Unexpectedly, our use of the participation measurements revealed that the proportion of IJs not participating (i.e., remaining in the center) across most species was lower and did not vary as much as has been reported in previous studies [5,18]. For example, even known ambusher species like *S. carpocapsae* [22,31,32] and intermediate foragers like *S. feltiae* [5,25] exhibited more than 50% of the population participating (Supplementary Figure S1 and Supplementary Figure S1A), and from species to species across all doses this appeared to be the case; with the exception of *S. riobrave* in response to 0.002 M prenol, which had less than 50% of the IJs participating in chemotaxis. It can be noted that there are some statistically significant differences between select species at each dose, but the magnitude of the differences was small; furthermore, at the 0.2 M dose, none of the species varied in the proportion of IJs not participating in the assay (Supplementary Figure S1). 

This high participation may be due in great part to the design of the assay. In particular, previous studies measuring participation have used a larger region, spanning the diameter of the plate across the center around the initial placement zone, while in our study, the “center” was limited to a 1 cm circle. We expected that there would be differences in the participation that correlated with a foraging strategy such that cruisers had a higher proportion of nematodes participating and that ambushers would have fewer nematodes participate in chemotaxis behavior. However, the dose–response participation values revealed that under no circumstances did these two species differ in their participation scores (based on the proportion remaining in the center).

Even when including the analyses of the proportion of the population of IJs within the blank quadrants, we still found that participation does not vary greatly among the tested species (Supplementary Figure S1B), indicating that this particular assay design affects the participation behavior and corrects for these disparities. This suggests that the use of PF127 media or assay design affects IJ participation values. This may be due to the increase in assay run time, the allowance of three-dimensional movement, or the effects of additional tactile feedback the nematodes experience while moving through the matrix. It could be a combination of any of these factors, or others we have not suggested here. More research utilizing PF127 in the studies of other nematodes species, and perhaps with different odors is needed to enhance our understanding of how the allowance of three-dimensional movement may affect the behavior of nematodes, such as EPNs and other parasitic species. 

## 5. Conclusions

In conclusion, we examined the response of EPNs to prenol using PF127 and found that they are repelled, just as they are in an agar petri dish arena. Furthermore, we used PF127 to study the effects of storage time on EPN chemotaxis behavior, and found that the effects are species specific, with *S. carpocapsae* experiencing a significant loss of repulsion behavior with increased storage time, while *S. glaseri* and *S. feltiae* did not. Our findings add support to the notion that PF127 media is an appropriate media for examining nematode behavior with distinct advantages over using agar petri dishes or soil/sand, though additional research is still needed to fully understand how this media may influence nematode behavior.

## Supplementary Materials

The following are available online at https://www.mdpi.com/2075-4450/11/8/457/s1, Figure S1: Proportion of the population within the center (initial placement zone) and the blank quadrants, Figure S2: An additional analysis of the participation data from the storage-time effect on chemotaxis (response to 2 M prenol), evaluating differences between the different species in regard to the proportion of the population that remained in the center (initial placement zone), Figure S3: Participation scores for dose–response assays, Figure S4: Effects of storage time on participation of *Steinernema* IJs responding to 2 M prenol.
