# Supplementary material for: EPNs Exhibit Repulsion to Prenol in Pluronic Gel Assays
Source: Insects. 2020 Jul 22;11(8):457. doi: 10.3390/insects11080457 (PMC7468948; doi:10.3390/insects11080457)
Supplement: Supplementary file 1 [file insects-11-00457-s001.pdf]

Supplementary Figures

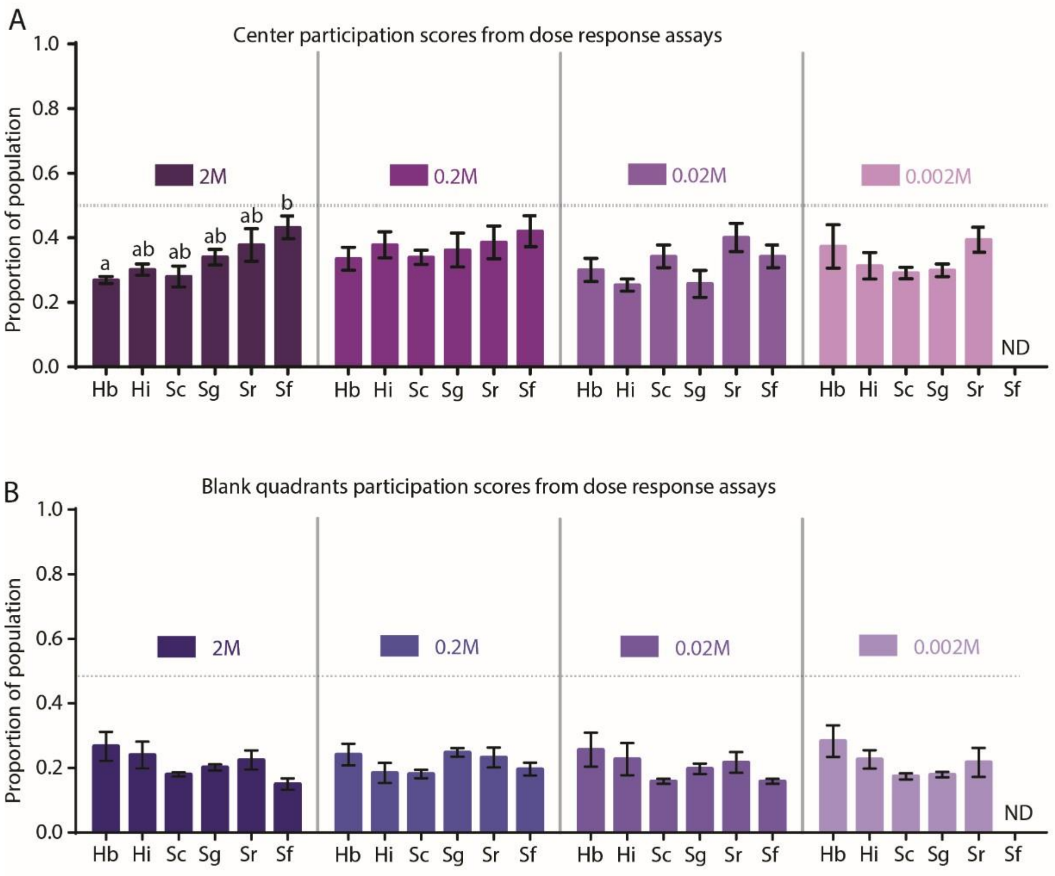

Supplementary Figure 1. Proportion of the population within the center (initial placement zone) and the blank quadrants. Participation data from the dose–response assays were analyzed for statistically significant differences between the species. *H. bacteriophora* (*Hb*), *H. indica* (*Hi*), *S. carpocapsae* (*Sc*), *S. glaseri* (*Sg*), *S. riobrave* (*Sr*) and *S. feltiae* (*Sf*). The notation ND indicates that no data were collected for Sf at the 0.002 M dose. (**A**) Proportion of the population that remained within the 1 cm circle—designated as the center region (initial placement zone)—for each species and dose tested. Statistical comparison between species for each dose revealed no differences between species in each dose, with the exception of the 0.2 M dose in which statistical differences were detected between *H. bacteriophora* and *S. feltiae*, with all other species exhibiting a participation score between (but not significantly different from) either of the two species. (**B**) Proportion of the population that moved to and were scored as being in the blank quadrants for each species and dose tested. Statistical analysis indicated that there was no difference between the species for any of the doses evaluated. Statistical analysis was done using a two-way ANOVA evaluation of all doses (additional details in methods). Statistical differences are at minimum $p < 0.05$. Bars represent mean with error bars representing SEM. Letters designate statistical differences, bars with the same letter are not statistically different. Bars with no letter designates that within that dose no statistically significant differences were detected.

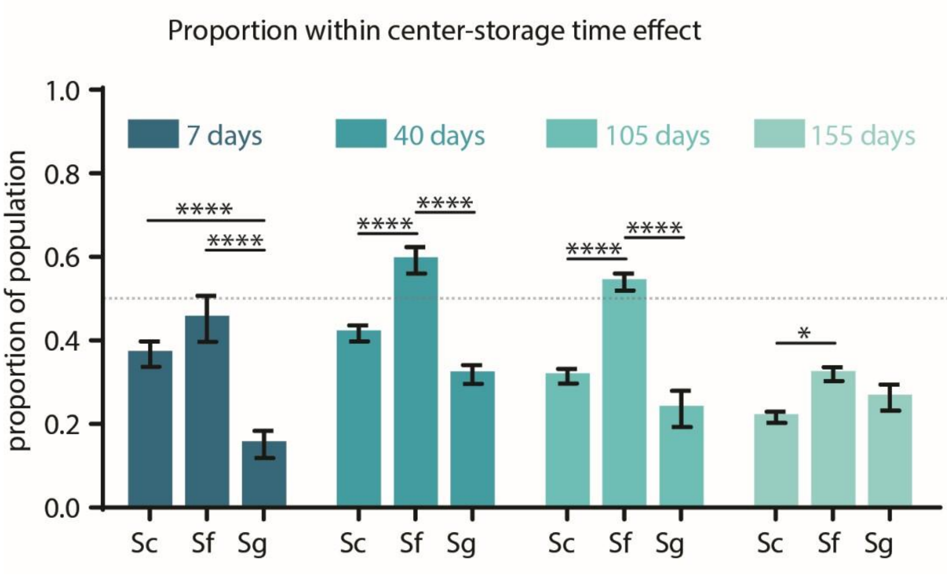

Supplementary Figure 2. An additional analysis of the participation data from the storage-time effect on chemotaxis (response to 2 M prenol), evaluating differences between the different species in regard to the proportion of the population that remained in the center (initial placement zone). For each time point of 7 days, 40 days, 105 days and 155 days, a comparison between the species within these time points was performed using a two-way ANOVA with Tukey's multiple comparisons test. ** $p < 0.01$, **** $p < 0.0001$.

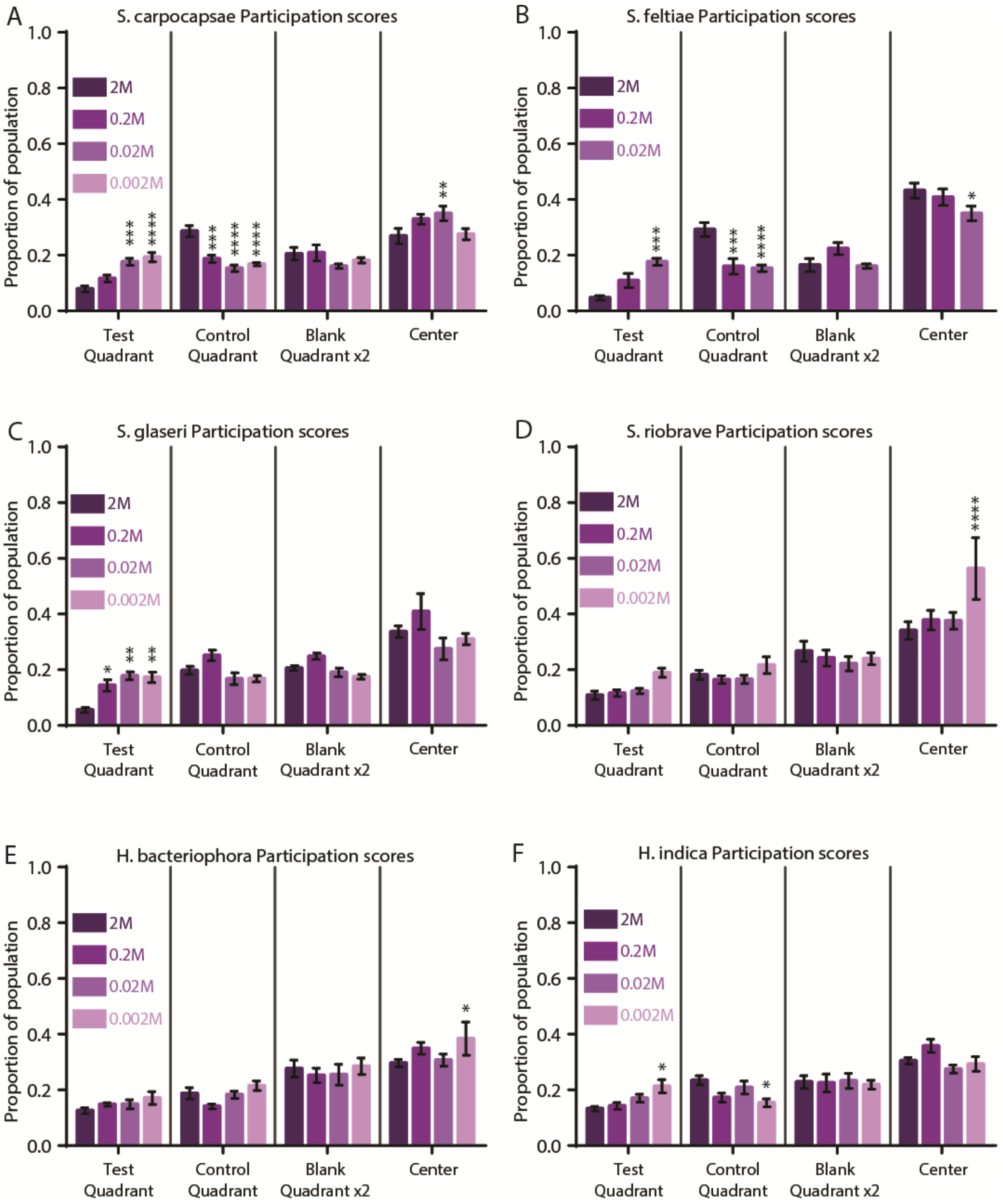

Supplementary Figure 3. Participation scores for dose–response assays. The scores reflect the participation of the entire population of IJs on the plate pertaining to the dose–response chemotaxis assays provided in figure 3. Participation scores of (**A**) *S. carpocapsae*, (**B**) *S. feltiae*, (**C**) *S. glaseri*, (**D**) *S. riobrave*, (**E**) *H. bacteriophora*, and (**F**) *H. indica.* Statistical analysis was performed using a two-way ANOVA with Dunnett's multiple comparisons post-test which compared all values to the response to 2 M; within the categories of *test, control, blank* and *center,* but not between these categories.

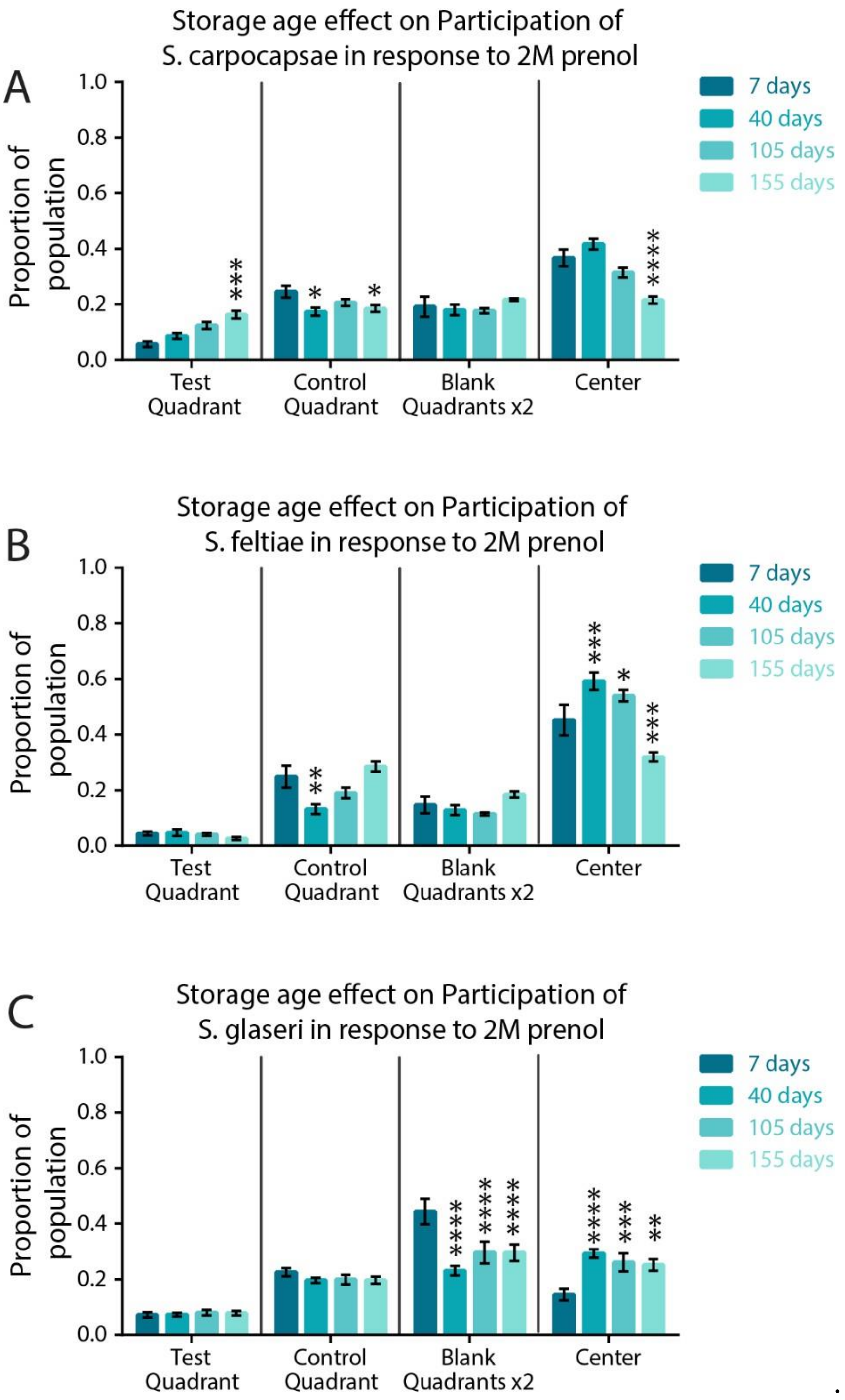

Supplementary Figure 4. Effects of storage time on participation of *Steinernema* IJs responding to 2 M prenol. Scores reflect the participation of the entire population of IJs on the plate pertaining to the chemotaxis index information provided in Figure 1. Participation values reflect the proportion of the population that was found within each quadrant and the center of the plate. IJs were stored at 15 °C for the specified durations (7 days, 40 days, 105 days, and 155 days), and their overall movements within the assay arena were recorded after 2 h for *S. glaseri*, 1 h for *S. feltiae*, and 6 h for *S. carpocapsae* before being scored. Participation values of (**A**) *S. carpocapsae*, (**B**) *S. feltiae*, and (**C**) *S. glaseri*. Statistical analysis for all assays was done using a two-way ANOVA with Dunnett's multiple comparisons post-test. The analysis was done within each category of *test*, *control*, *blank*, and *center*; but not between the categories. Comparisons of each value were made to the earliest time point post-collection (7 days).
